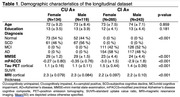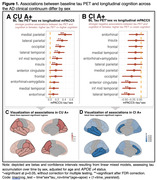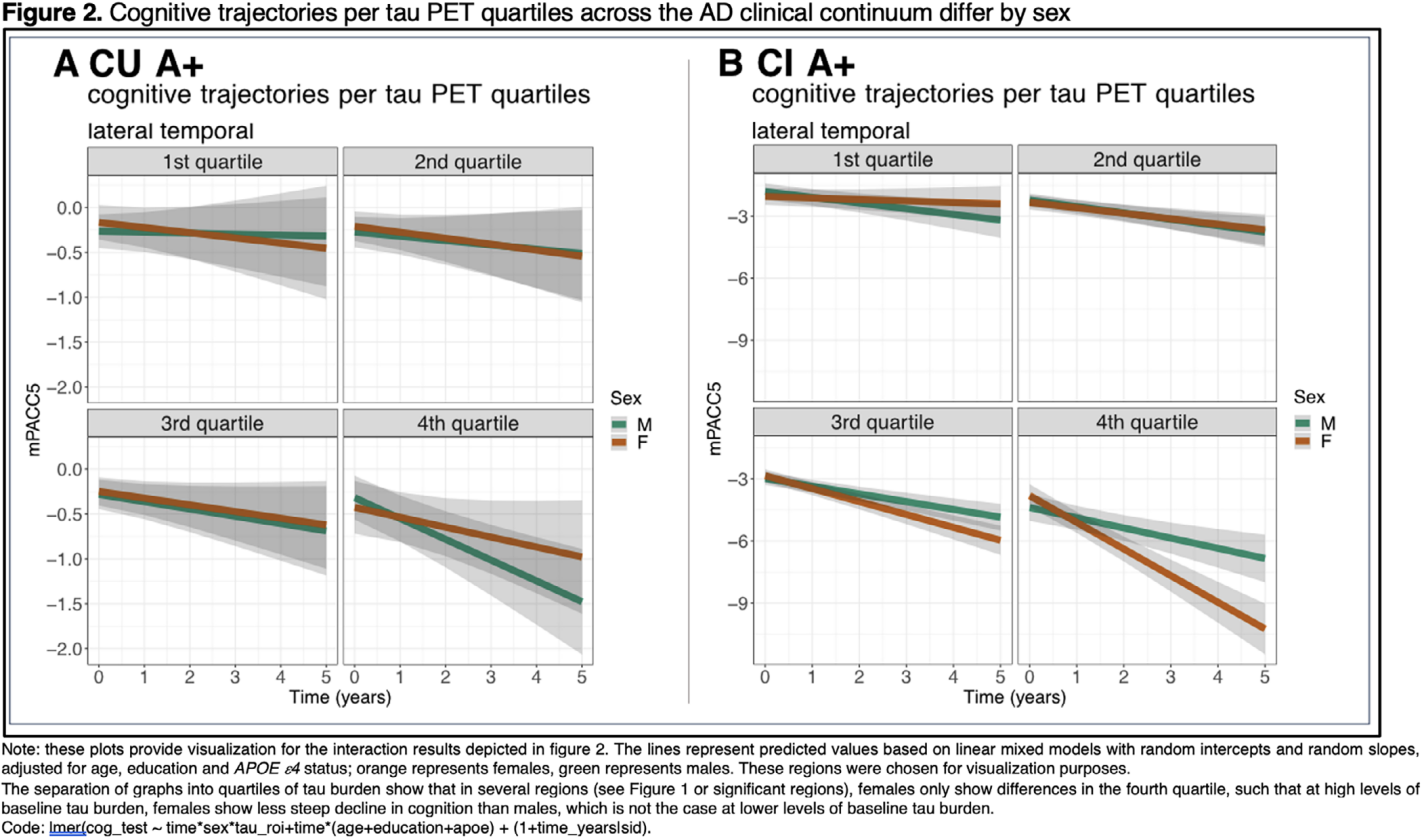# Associations between tau accumulation and cognitive decline across the AD continuum differ by sex

**DOI:** 10.1002/alz.091527

**Published:** 2025-01-09

**Authors:** Ellen Hanna Singleton, Niklas Mattsson‐Carlgren, Alexa Pichet Binette, Olof Strandberg, Erik Stomrud, Rik Ossenkoppele, Oskar Hansson

**Affiliations:** ^1^ Clinical Memory Research Unit, Department of Clinical Sciences, Lund University, Lund Sweden; ^2^ Clinical Memory Research Unit, Lund University, Lund Sweden; ^3^ Alzheimer Center Amsterdam, Department of Neurology, Amsterdam Neuroscience, Vrije Universiteit Amsterdam, Amsterdam UMC, Amsterdam Netherlands

## Abstract

**Background:**

female sex has been associated with faster tau accumulation over time and greater resilience in Alzheimer’s disease. However, relatively few studies have examined regional tau accumulation rates and cognitive resilience in a large amyloid‐positive cohort.

**Method:**

We included n=761 amyloid‐positive (A+) participants from the Swedish BioFINDER‐2 study who had undergone (longitudinal) tau PET and cognitive testing, of whom n=253 were cognitively unimpaired (CU A+) and n=508 were cognitively impaired (CI A+). [^18^F]RO948‐tau‐PET SUVR within entorhinal, medial temporal, neocortical temporal, medial parietal, lateral parietal, occipital, frontal, anterior cingulate, insula and cortical composite regions was quantified. Cognition was assessed with the modified preclinical Alzheimer’s disease cognitive composite (mPACC5). Linear mixed models were fitted to assess the effect of the interaction between sex and baseline tau on cognition over time, adjusting for age, education and APOE e4 status.

**Result:**

Demographic characteristics are shown in Table 1. Models assessing the effect of the interaction between baseline tau and sex on cognitive decline showed significant positive associations between multiple tau regions in CU A+ and longitudinal mPACC5 (medial parietal: 0.39±0.10, p=0.0005, lateral parietal: 0.31±0.08, p=0.0005, occipital: 0.26±0.07, p=0.0005, lateral temporal: 0.23±0.07, p=0.006, inferior middle temporal: 0.18±0.07, p=0.03, insula: 0.17±0.07, p=0.04, Figure 1), while models showed significant negative associations between some tau regions in CI A+ and longitudinal mPACC5 (lateral temporal: ‐0.38±0.11, p=0.007, inferior middle temporal: ‐0.35±0.11, p=0.01, anterior cingulate: ‐0.35±0.13, p=0.03, Figure 1). Assessing cognitive trajectories by sex per quartiles of baseline tau burden revealed that females show similar cognitive trajectories to males in the 1^st^‐3^rd^ quartiles of tau burden, but seem to show less cognitive decline in the 4^th^ quartile of tau burden in CU, while in CI, females show a steeper decline than males (Figure 2).

**Conclusion:**

Our results suggest that cognitively unimpaired A+ females exhibit greater cognitive resilience, while cognitively impaired A+ females exhibit steeper cognitive decline than males in the face of tau pathology, and that these effects occur particularly at high levels of tau pathology.